# Single nucleolus precursor body formation in the pronucleus of mouse zygotes and SCNT embryos

**DOI:** 10.1371/journal.pone.0202663

**Published:** 2018-08-20

**Authors:** Hirohisa Kyogoku, Teruhiko Wakayama, Tomoya S. Kitajima, Takashi Miyano

**Affiliations:** 1 Graduate School of Agricultural Science, Kobe University, Kobe, Japan; 2 Laboratory for Chromosome Segregation, Center for Developmental Biology, RIKEN, Kobe, Japan; 3 Laboratory for Chromosome Segregation, RIKEN Center for Biosystems Dynamics Research, Kobe, Japan; 4 Faculty of Life and Environmental Sciences, University of Yamanashi, Kofu, Japan; Nanjing Agricultural University, CHINA

## Abstract

Mammalian oocytes and zygotes have nucleoli that are transcriptionally inactive and structurally distinct from nucleoli in somatic cells. These nucleoli have been termed nucleolus precursor bodies (NPBs). Recent research has shown that NPBs are important for embryonic development, but they are only required during pronuclear formation. After fertilization, multiple small NPBs are transiently formed in male and female pronuclei and then fuse into a single large NPB in zygotes. In cloned embryos produced by somatic cell nuclear transfer (SCNT), multiple NPBs are formed and maintained in the pseudo-pronucleus, and this is considered an abnormality of the cloned embryos. Despite this difference between SCNT and normal embryos, it is unclear how the size and number of NPBs in pronuclei is determined. Here, we show that in mouse embryos, the volume of NPB materials plays a major role in the NPB scaling through a limiting component mechanism and determines whether a single or multiple NPBs will form in the pronucleus. Extra NPB- and extra MII spindle-injection experiments demonstrated that the total volume of NPBs was maintained regardless of the pronucleus number and the ratio of pronucleus/NPB is important for fusion into a single NPB. Based on these results, we examined whether extra-NPB injection rescued multiple NPB maintenance in SCNT embryos. When extra-NPBs were injected into enucleated-MII oocytes before SCNT, the number of NPBs in pseudo-pronuclei of SCNT embryos was reduced. These results indicate that multiple NPB maintenance in SCNT embryos is caused by insufficient volume of NPB.

## Introduction

The nucleolus formed in the germinal vesicle (GV) of fully-grown mammalian oocytes does not contain DNA [1]. This nucleolus have no transcriptional activity and distinct structure from the nucleoli in somatic cells, and has been termed a nucleolus precursor body (NPB) [2]. After GV breakdown during oocyte maturation, the NPBs disassemble and their materials were scattered into the cytoplasm, and upon fertilization male and female pronuclei formed with NPBs in zygotes. The NPBs formed in zygote are also transcriptionally inactive and morphologically similar to the oocyte NPBs in GV [2]. Previous studies have shown that the oocyte NPBs are required for early embryonic development [3–5]. When the NPBs were removed micro-surgically from the mammalian oocytes at the GV stage, the enucleolated oocytes were able to mature to metaphase II (MII) and to be fertilized. However, the enucleolated oocytes neither formed NPBs in the pronucleus nor developed to blastocysts after fertilization.

When oocyte NPBs were injected into enucleolated and matured oocytes and these oocytes were then fertilized, they formed pronuclei containing NPBs and developed to blastocysts and after transfer to recipients, live pups were obtained [3]. On the other hand, when the NPBs were injected into previously enucleolated and fertilized zygotes at the pronuclear stage, the development rates of blastocysts and full-term were severely reduced [4]. We have also shown that NPBs are not required for early embryonic development after the late pronuclear formation stage [5]. When NPBs were removed from both of pronuclei at late pronuclear formation stage, the enucleolated zygotes developed to full-term [5]. Combining these results, it can be concluded that the NPB is only required during pronuclear formation.

Because the NPB is a non-membrane-bound structure and its active liquid-like behavior has been reported in Xenopus [6] and mouse oocytes [7], the centrifugation or artificial contact allowed the NPBs to fuse. With centrifugation of two-cell pig embryos, multiple NPBs in each nucleus fused into a single NPB [8]. Moreover, isolated oocyte NPBs fused efficiently when NPBs were contacted in vitro [7]. In addition, when NPBs from other oocytes were injected into the cytoplasm of previously enucleolated oocytes, they quickly disappeared but were later reassembled in the GVs [9].

After fertilization, numerous small NPBs are typically formed in the pronucleus. As the cell cycle progresses, these NPBs fuse to form a single larger structure tethering the centromeres to their surface [10–12]. It has been suggested that the number and size of NPBs in pronuclei is important for embryonic development in human embryos [13, 14]. Moreover, the cloned embryos produced by somatic cell nuclear transfer (SCNT) exhibit abnormalities including multiple NPB maintenance in pseudo-pronuclei [15]. However, it has been unclear how the size and number of NPBs in pronuclei is determined.

Here, we generate various types of embryos with different NPB volume / pronucleus ratios and demonstrate that multiple NPB maintenance is caused by an insufficient volume of NPBs. Then, we conduct extra-NPB injection experiments to determine the effect of the NPBs on abnormal multiple NPB maintenance in SCNT mouse embryos. The results show that injection of extra-NPBs induces formation of large NPBs and prevents the multiple NPB maintenance in the pseudo-pronuclei of cloned embryos.

## Materials and methods

### Animals

B6D2F1 (C57BL/6 × DBA/2) mice, aged eight- to ten-weeks, were used to produce oocytes. The surrogate pseudopregnant females used as embryo transfer recipients (see below) were ICR strain mice mated with vasectomized males of the same strain. B6D2F1 and ICR mice were purchased from Japan SLC Inc (Hamamatsu, Japan). All animal experiments conformed to the Guide for the Care and Use of Laboratory Animals and were approved by the Institutional Committee of Laboratory Animal Experimentation of the RIKEN Center for Biosystems Dynamics Research.

### Chemicals

All chemicals were purchased from Sigma-Aldrich (St. Louis, MO, USA) unless otherwise indicated.

### Collection and culture of oocytes

Collection and culture of oocytes was carried out as described previously [5, 16]. Eight- to ten-week-old female mice were injected with 5 IU of equine chorionic gonadotropin (eCG, ASKA Pharmaceutical, Tokyo). Fully-grown oocytes at the GV stage were collected 44 h after injection. They were released directly into M2 medium supplemented with 200 nM 3-isobutyl-1-metyl-xanthine (IBMX, Sigma) from antral follicles, and their cumulus cells were removed by pipetting. The oocytes were then cultured for at least 30 min in αMEM (Gibco, Carlsbad, CA, USA) with 10% (v/v) fetal bovine serum (FBS; ICN Biomedicals, Inc., Aurora, OH, USA), 1 mM sodium pyruvate, 100 IU/ml penicillin, 0.1 mg/ml streptomycin sulfate, and 200 nM IBMX at 37°C under an atmosphere of 5% CO_2_ in air until used.

Mature oocytes were collected from the oviducts of eight- to ten-week-old female mice that had been induced to superovulate with 5 IU of eCG followed by 5 IU of human chorionic gonadotropin (hCG; ASKA Pharmaceutical) 48 h later. Cumulus-oocyte complexes (COCs) were collected from the oviducts approximately 16 h after hCG injection. COCs were placed in HEPES-buffered Chatot, Ziomek, and Bavister medium (H-CZB) [17] and treated with 0.1% (w/v) bovine testicular hyaluronidase. After several minutes, the cumulus-free oocytes were washed twice and then moved to KSOM (Merck Millipore, Billerica, MA, USA). Mature metaphase II (MII) oocytes were subjected to ICSI and SCNT.

### Collection of oocyte NPBs

Collection of NPBs was carried out as described previously [5, 18]. Briefly, each oocyte was held by a holding pipette (inner diameter 10 μm, angle 30°) at the 9 o’clock position, and then the zona pellucida was punctured with a square-ended injection pipette and piezo pulses using a micromanipulator (Narishige Group, Tokyo) equipped with a PIEZO drive (PNAS-CT150; Prime Tech, Ibaragi, Japan). After penetration through the zona pellucida, the injection pipette was positioned against the membrane of the GV. Thereafter, gentle suction was applied, which resulted in suction of the NPB into the tip of the pipette. The injection pipette was then slowly withdrawn from the cytoplasm. When the pipette opening with the captured NPB was just outside the cell membrane, additional suction was applied. Then the NPB penetrated the membrane of the GV, leaving the entire nuclear content of the GV within the nuclear envelope. Previous reports showed that NPBs were able to remain in the form of so-called nucleoloplasts, i.e., they were enclosed with a small volume of nucleoplasm surrounded by the oocyte plasma membrane (S1A Fig) [7] and they have a potential for embryonic development after 24 h maintenance [3, 4]. However, they were fragile, and after breaking the plasma membrane they immediately disappeared into the medium (S1A Fig). Even the nucleoloplasts disappeared after a short time (S1B Fig). So, we generated a system to better maintain the NPBs. When NPBs were placed in the culture medium supplemented with a high-molecular-weight compound (10% polyvinylpyrrolidone [PVP]), their structure was maintained at least 48 h (S1B Fig). The collected NPBs from GV oocytes were kept in the PVP-supplemented medium before use for the NPB injection experiments (within 2h).

### NPB injection

NPB injection was carried out as described previously [18]. Collected NPBs from GV oocytes were injected into some of the MII oocytes (S1C Fig). The injection of NPBs was performed in a manner similar to that of ICSI [19]. Each oocyte was held by a holding pipette at the 9 o’clock position. The zona pellucida was punctured by several applications of piezo-pulse. Next, one NPB from a GV oocyte, which had been aspirated into a glass micropipette, was ejected into the cytoplasm of the oocyte. The injection pipette was then slowly withdrawn. The MII recipient oocytes were cultured for 1 h before ICSI or SCNT. A group of sham-operated controls was established separately by injecting the oocytes with a small volume of medium.

### Intra-cytoplasmic sperm injection

ICSI with sperm heads was carried out as described previously [19] and injected oocytes were cultured in KSOM. The number of NPBs in pronuclei was counted at 10 h post-ICSI. After pictures were obtained with a digital camera (DP21; Olympus), the diameters of all NPBs were measured by software (Fiji; https://fiji.sc/) [20] and the total volume of NPBs was acquired.

### Injection of MII spindles and parthenogenetic activation

Groups of MII oocytes were transferred into droplets of H-CZB containing 5 mg/ml cytochalasin B (CB) on the microscope stage for collection of the MII spindle. Oocytes undergoing microsurgery were held with a holding pipette and a hole was made in the zona pellucida via the application of several piezo-pulses using an enucleation pipette. The MII spindle was aspirated into the pipette with a minimal volume of ooplasm. For MII spindle injection, a donor MII spindle was injected into another intact MII oocyte. These reconstructed oocytes were kept in the incubator until stimulation. Reconstructed oocytes were stimulated parthenogenetically using 10 mM SrCl_2_ in KSOM containing 2 mM ethylene glycol tetraacetic acid (EGTA) in the presence of 5 μM latrunculin A for 10 h, and then fixed for immunofluorescence staining.

### Somatic cell nuclear transfer (SCNT)

From MII oocytes, the chromosome–spindle complexes were removed as described above, and the resulting enucleated oocytes were transferred into KSOM. For nuclear injection, donor cumulus cells were gently aspirated in and out of the injection pipette to broken plasma membranes. Each nucleus was injected into an enucleated oocyte, and these reconstructed oocytes were kept in the incubator until activation. Reconstructed oocytes were activated parthenogenetically using SrCl_2_ for 10 h as described above, and some of them were cultured in KSOM until embryo transfer. For the other reconstructed oocytes, the number of NPBs in pseudo-pronuclei was counted at 10 h post-parthenogenetic activation. After pictures were obtained using a digital camera (DP21), the diameters of all NPBs were measured by software (Fiji) and the total volume of NPBs was acquired.

### Embryo transfer

Embryo Transfer was carried out as described previously [5]. Embryos at the two-cell stage were transferred at 0.5 days post-coitum into the oviducts of pseudopregnant ICR strain female mice that had been mated with a vasectomized male the night before transfer. At 18.5 days post-coitum, the offspring were delivered by Caesarean section because mother sometimes eats their pups. Surviving pups were fostered to an ICR foster mother that had given birth on the same day.

### Fluorescence microscopy

Immunofluorescence staining was carried out as described previously [5]. Pronucleus stage embryos and blastocysts were collected at 10 h and at 3.5 days after ICSI or strontium activation, respectively. They were fixed in 2% paraformaldehyde in phosphate-buffered saline (PBS)-polyvinyl alcohol (PVA) (pH 7.4) for 30 min. After samples were blocked and permeabilized in PBS-PVA containing 1 mg/ml BSA (PBS-PVA-BSA) and 0.1% Triton X-100 (Nakalai Tesque Inc., Kyoto, Japan), the embryos and blastocysts were incubated with the appropriate primary antibodies at 4°C overnight, washed several times in PBS-PVA-BSA and incubated with secondary antibodies for 120 min at room temperature. For pronucleus stage embryo staining, DNA was counterstained with 40 μg/ml of Hoechst 33342. Finally, the embryos were washed and transferred to BSA–PVA for imaging with a Zeiss LSM780 confocal microscope. For blastocysts staining, they were mounted on glass slides with ProLong Gold Antifade Reagent with DAPI (Molecular Probes, Eugene, OR, USA) and observed under a confocal laser scanning microscope (FV1000; Olympus Co., Tokyo). The following primary antibodies were used: rabbit polyclonal anti-Oct3/4 antibody (1:200, sc-9081; Santa Cruz Biotechnology Inc., Santa Cruz, CA, USA); mouse monoclonal anti-Cdx2 antibody (1:200, AM392; BioGenex, Fremont, CA, USA); and mouse anti-Histone H3 antibody (1:200, ab195277, ab62706; Abcam, Cambridge, UK). The secondary antibodies were Alexa Fluor 488 Goat Anti-Mouse IgG (H+L) (A11029), Alexa Fluor 488 Goat Anti-Rabbit IgG (H+L) (A11034), Alexa Fluor 568 Goat Anti-Rabbit IgG (H+L) (A11011), and Alexa Fluor 555 Goat Anti-mouse IgG (H+L) (A21424) (1:400; Molecular Probes).

### Live cell imaging

Live Cell Imaging was carried out as described previously [5]. For detecting NPBs and chromosomes, enhanced green fluorescent protein (EGFP) coupled with nucleoplasmin 2 (NPM2) (EGFP-NPM2) and monomeric cherry (mCherry) fused with histone H2B (H2B-mCherry) were used, respectively. EGFP-NPM2 and H2B-mCherry plasmid (pCS2+) were provided by S. Ogushi (Kyoto University, Japan).

mRNA injection into oocytes was performed as described previously [21]. Briefly, each mRNA was diluted to 50 ng/μl. Collected MII oocytes were transferred to droplets of H-CZB medium in the chamber and a few picoliters of mRNA solution was injected into the oocyte cytoplasm using a micromanipulator equipped with a PIEZO drive with a glass micropipette (1–3 μm in diameter). After mRNA injection, the oocytes were manipulated (NPB injection, ICSI and SCNT) as described above.

The embryos were transferred to drops of CZB medium on a glass-bottomed dish, placed in an incubator (MI-IBC; Tokai Hit, Shizuoka, Japan) on the microscope stage and incubated at 37°C under 5% CO_2_ in air. The devices used for the imaging were as described previously [22]. For time-lapse observations, images were taken over 10 h at 2-min intervals. At each time point, 51 fluorescent images were taken 2 μm apart along the Z-axis for optical sectioning. Time-lapse observations of ICSI and SCNT embryos were started just after ICSI and Sr activation, respectively.

### Statistical analysis

Graphs were generated and statistical analyses were performed using Excel and GraphPad Prism. The frequencies of embryos at each stage of development and the percentages of single NPB formation in pronucleus were analyzed using the chi-square test. Differences in the mean (±SEM) volumes of NPBs were analyzed by the Two-tailed, unpaired Student’s *t*-test. Sample sizes, statistical tests and p-values are indicated in figures and figure legends.

## Results

### The volume of NPBs was determined by the amount of their components

**Fig 1:**
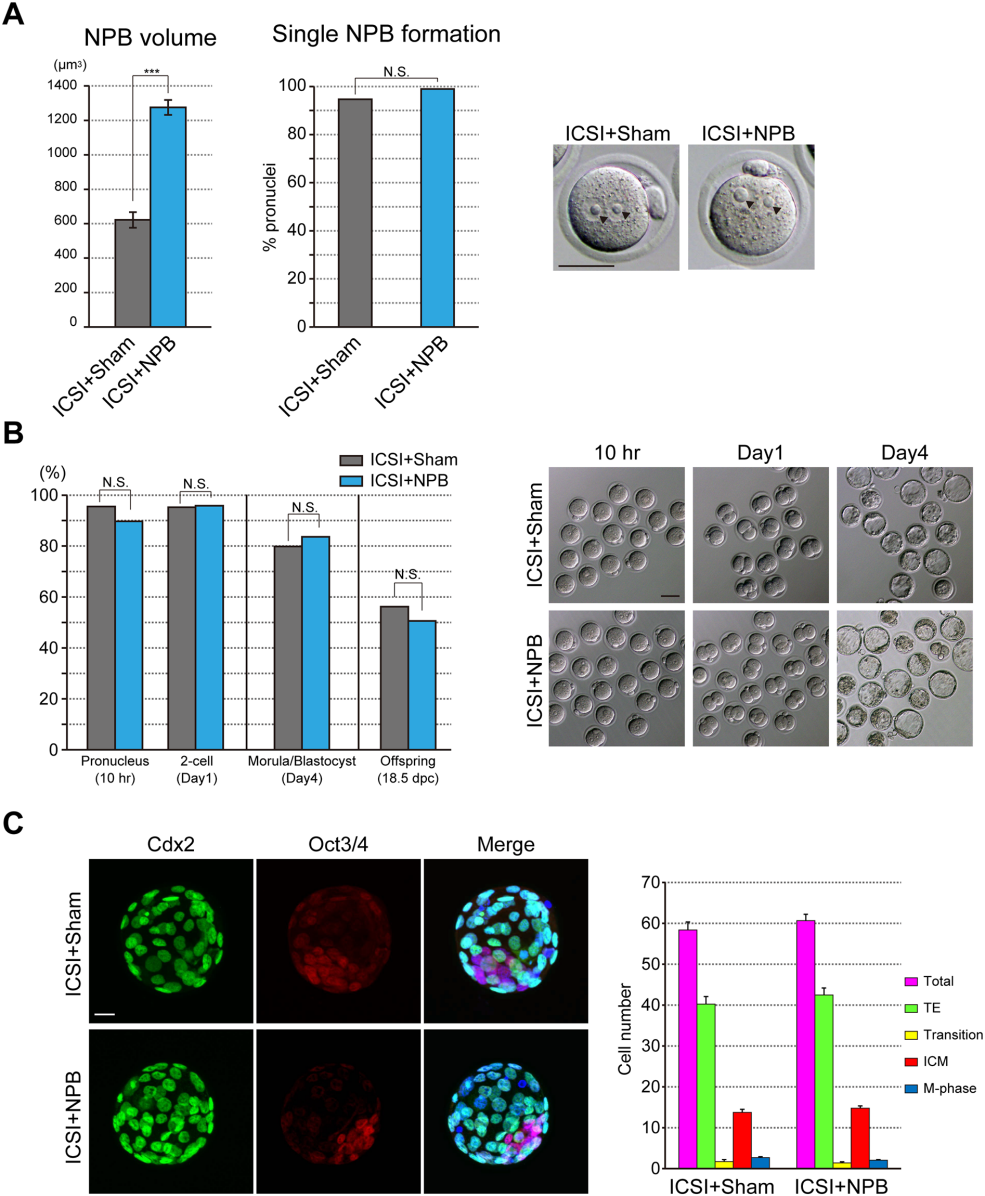
NPB volume and development of extra-NPB-injected embryos. (A) NPB volume and single NPB formation in pronuclei of extra-NPB-injected ICSI (ICSI+NPB) embryos. The total volumes of NPBs were analyzed in ICSI+Sham (n = 18) and ICSI+NPB (n = 61) zygotes. Bars represent the mean ± SEM. Two-tailed, unpaired Student’s *t*-test was performed. The percentages of single NPB formation in pronucleus were analyzed in ICSI+Sham (n = 378) and ICSI+NPB (n = 494) pronuclei. Chi-square test was performed. ***P<0.001: not significant (N.S.). Arrowheads indicate NPBs in pronuclei. The scale bar is 50 μm. (B) In vitro and in vivo development of ICSI+NPB embryos. The rates of embryos that formed pronuclei and developed to the two-cell stage (ICSI+Sham: n = 221; ICSI+NPB: n = 213), and development to the morula/blastocyst stage (ICSI+ Sham: n = 99; ICSI+NPB: n = 104) were analyzed. The rates of full-term development were analyzed after the transfer of two-cell-stage embryos into surrogate mothers (ICSI+Sham: n = 105/8 and ICSI+NPB: 81/7; embryos/recipients). Chi-square test was performed. not significant (N.S.). The scale bar is 50 μm. (C) Cell numbers and cell types of blastocysts were compared between ICSI+Sham embryos (n = 46) and ICSI+NPB embryos (n = 52). Cell numbers of the trophectoderm (TE) and inner cell mass (ICM) were counted after immunostaining for Cdx2 (green) and Oct3/4 (red), respectively. DAPI staining marks chromatin in blue. Cdx2/Oct3/4 doubly-positive cells (Transition) were counted on merged images. Metaphase cells (DAPI staining only) were counted on triple-merged images. The scale bar is 20 μm. Error bars in the graph indicate the standard error of the mean. Two-tailed, unpaired Student’s *t*-test was performed.

To investigate the NPB scaling in pronuclei, we generated embryos that were injected with NPBs at the MII stage and then subjected to ICSI (ICSI+NPB). Collected NPBs from GV oocytes placed in the culture medium supplemented with 10% PVP maintained their structure (S1A and S1B Fig). Then, the NPBs were injected into the cytoplasm of MII oocytes (S1C Fig). The injected NPBs were disassembled in the oocyte cytoplasm and reassembled in pronuclei after ICSI. In the ICSI+NPB embryos, the volume of NPBs in pronuclei was increased approximately twofold (Fig 1A; ICSI+Sham: 621.7±45.4μm^3^; ICSI+NPB: 1274.8±43.5μm^3^), although most of the pronuclei had a single NPB in both types of embryos (Fig 1A).

Next, we examined the effect of the large-volume NPBs on the embryonic development. In both NPB-injected embryos (ICSI+NPB) and control embryos (ICSI+Sham), the total percentages of embryos forming pronuclei 10 h after ICSI (ICSI+Sham: 95.5%; ICSI+NPB: 89.7%) and developing to two-cell embryos (ICSI+Sham: 95.2%; ICSI+NPB: 95.8%) were similar (Fig 1B). The embryos forming pronuclei were further cultured to examine their development. There was no significant difference in the morula/blastocyst formation rate (ICSI+Sham: 79.8%; ICSI+NPB: 83.7%; Fig 1B). The morphology of developing ICSI+NPB embryos was also similar to that of control ICSI+Sham embryos (Fig 1B). We characterized the blastocysts that originated from ICSI+NPB embryos by immunostaining. In mouse blastocysts, the transcription factor Oct3/4 is restricted to the inner cell mass (ICM), and the trophectoderm (TE) is marked by Cdx2 [23]. The numbers of ICM and TE cells in the blastocysts originating from ICSI+NPB embryos were similar to those in ICSI+Sham embryos (Fig 1C). After transfer of two-cell-stage ICSI+NPB embryos into surrogate mothers (Fig 1B), there were no significant differences in the final birth rates between ICSI+Sham (56.2%) and ICSI+NPB embryos (50.6%). Previous studies reported that the oocyte NPBs were required for early embryonic development after fertilization [3, 5]. Our present data show that extra-NPB injection had no effect on early embryonic development after fertilization. Collectively, these findings indicate that ICSI+NPB embryos have a sufficient volume of NPB for embryonic development.

### NPB volume / pronucleus ratio is important for single NPB formation

**Fig 2:**
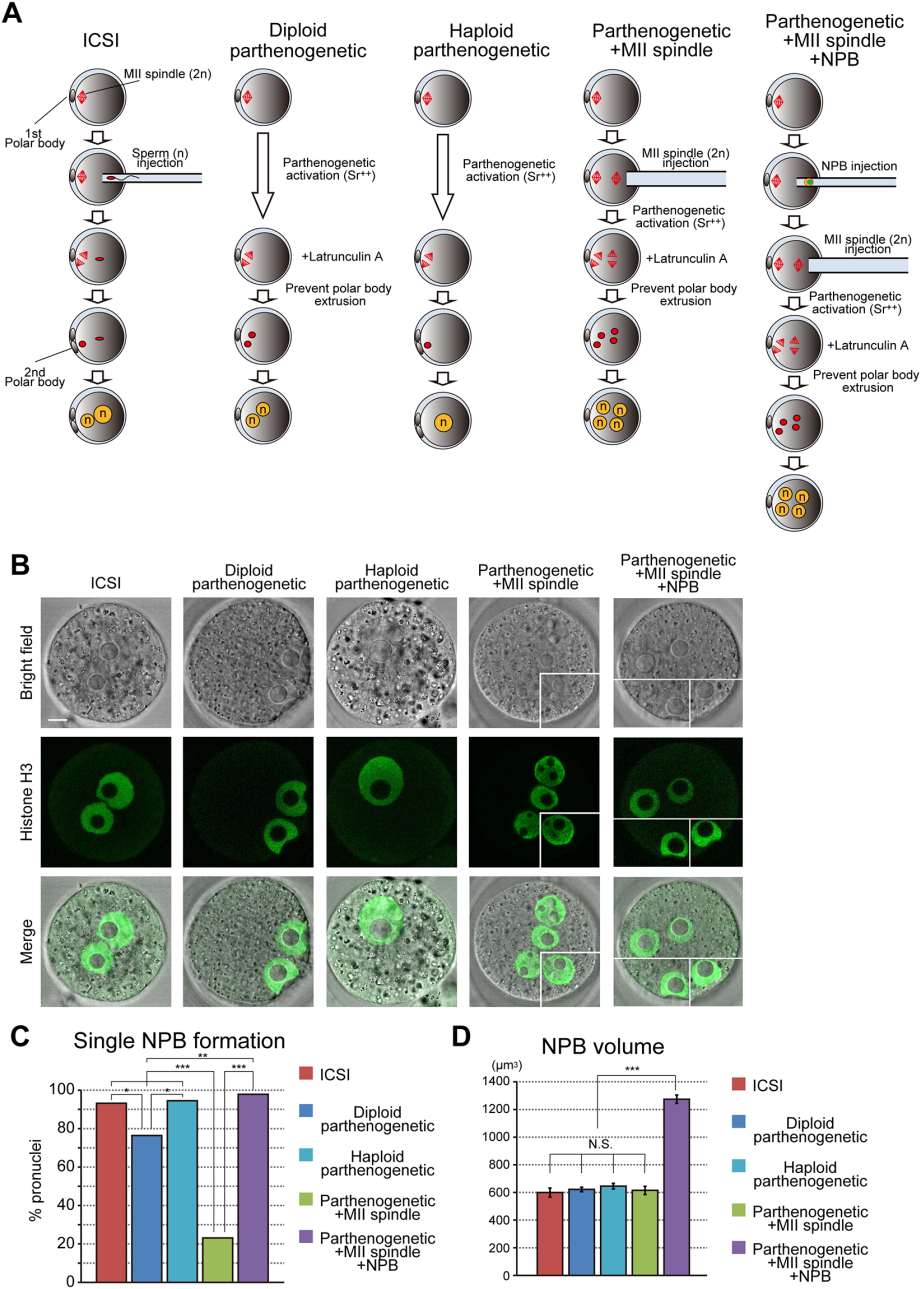
NPB volume affects the number of NPBs in the pronucleus. (A) Experimental scheme. Oocytes were injected with an extra-MII spindle and then subjected to parthenogenetic activation (Parthenogenetic+MII spindle). An extra-NPB was injected into the cytoplasm of MII oocytes before extra-MII-spindle injection (Parthenogenetic+MII spindle+NPB). Polar body extrusion was prevented by latrunculin A treatment. The volume of NPB per pronucleus was doubled in Haploid parthenogenetic embryos and halved in Parthenogenetic+MII spindle embryos, whereas each pronucleus had a haploid number of chromosomes. (B) Histone H3 Immunostaining and counting of NPBs in Parthenogenetic+MII spindle+NPB embryos. Embryos were fixed at 10 h after ICSI or parthenogenetic activation for immunostaining with histone H3 (green). Images of Parthenogenetic+MII spindle and Parthenogenetic+MII spindle+NPB embryos were combined with 2–3 different single Z slice images in the same embryo. The scale bar is 10 μm. (C) Single NPB formation in embryos with different NPB/pronucleus ratios. The percentages of pronuclei with single NPB formation were analyzed in ICSI (n = 110), Diploid parthenogenetic (n = 44), Haploid parthenogenetic (n = 34), Parthenogenetic+MII spindle (n = 108) and Parthenogenetic+MII spindle+NPB (n = 47) pronuclei. Chi-square test was performed. *P<0.05, **P<0.01, ***P<0.001. (D) Total NPB volumes in different NPB/pronucleus ratio embryos. The total volumes of NPBs were analyzed in ICSI (n = 13), Diploid parthenogenetic (n = 42), Haploid parthenogenetic (n = 36), Parthenogenetic+MII spindle (n = 17) and Parthenogenetic+MII spindle+NPB embryos (n = 9). Bars represent the mean ± SEM. Two-tailed, unpaired Student’s *t*-test was performed. ***P<0.001: not significant (N.S.).

To investigate the relationship between the number and volume of NPBs per pronucleus, we generated embryos which had doubled and halved volumes of NPB per pronucleus. Parthenogenetic activation was used to generate the embryos with a doubled volume of NPB per pronucleus. After second polar body emission, these embryos contained one pronucleus which had a haploid number of chromosomes (Fig 2A). To generate the embryos with a halved volume of NPB per pronucleus, MII-stage oocytes were injected with an extra-MII spindle and then parthenogenetically activated. These oocytes were treated with latrunculin A to prevent the emission of the second polar body, resulting in embryos containing four pronuclei, each of which had a haploid number of chromosomes (Fig 2A). We immunostained the embryos to analyze the number and volume of NPBs (Fig 2B–2D). In the control ICSI and diploid parthenogenetic embryos, most of the pronuclei had a single NPB (93% and 76%, respectively, Fig 2C). Moreover, the haploid parthenogenetic embryos, which had a doubled volume of NPB per pronucleus, formed a single pronucleus with a single NPB (94%, Fig 2C). However, in the embryos injected with an extra-MII spindle (Parthenogenetic+MII spindle), most of the pronuclei contained multiple NPBs (77%, Fig 2C). To determine whether the multiple NPB maintenance was caused by the ratio of NPB volume / pronucleus, an extra-NPB was injected into each of the MII oocytes before extra-MII-spindle injection (Parthenogenetic+MII spindle+NPB, Fig 2A). In these embryos, which had the same ratio of NPB volume / pronucleus as diploid parthenogenetic or ICSI embryos, multiple NPB maintenance was rescued (98%, Fig 2C). These results indicate that the ratio of NPB volume / pronucleus determines whether single or multiple NPBs will form in the pronucleus.

The total volumes of NPBs in pronuclei of ICSI and diploid parthenogenetic embryos which had two pronuclei were similar (Fig 2D; ICSI: 599.2±32.2μm^3^; Diploid parthenogenetic: 622.0±16.4μm^3^). Moreover, in the embryos which had half (Haploid parthenogenetic) and double (Parthenogenetic+MII) the numbers of pronuclei, the total volumes of NPBs in pronuclei were also similar (Fig 2D; Haploid parthenogenetic: 645.7±20.1μm^3^; Parthenogenetic+MII spindle: 614.5±29.5μm^3^). However, in the Parthenogenetic+MII spindle+NPB embryos, the total volume of NPB in pronuclei was increased approximately twofold (Fig 2D; Parthenogenetic+MII spindle+NPB: 1273.6±30.2μm^3^). These results indicate that the total NPB volume per embryo is maintained by the amount of NPB materials.

### Single NPB formation in SCNT embryos after extra-NPB injection

**Fig 3:**
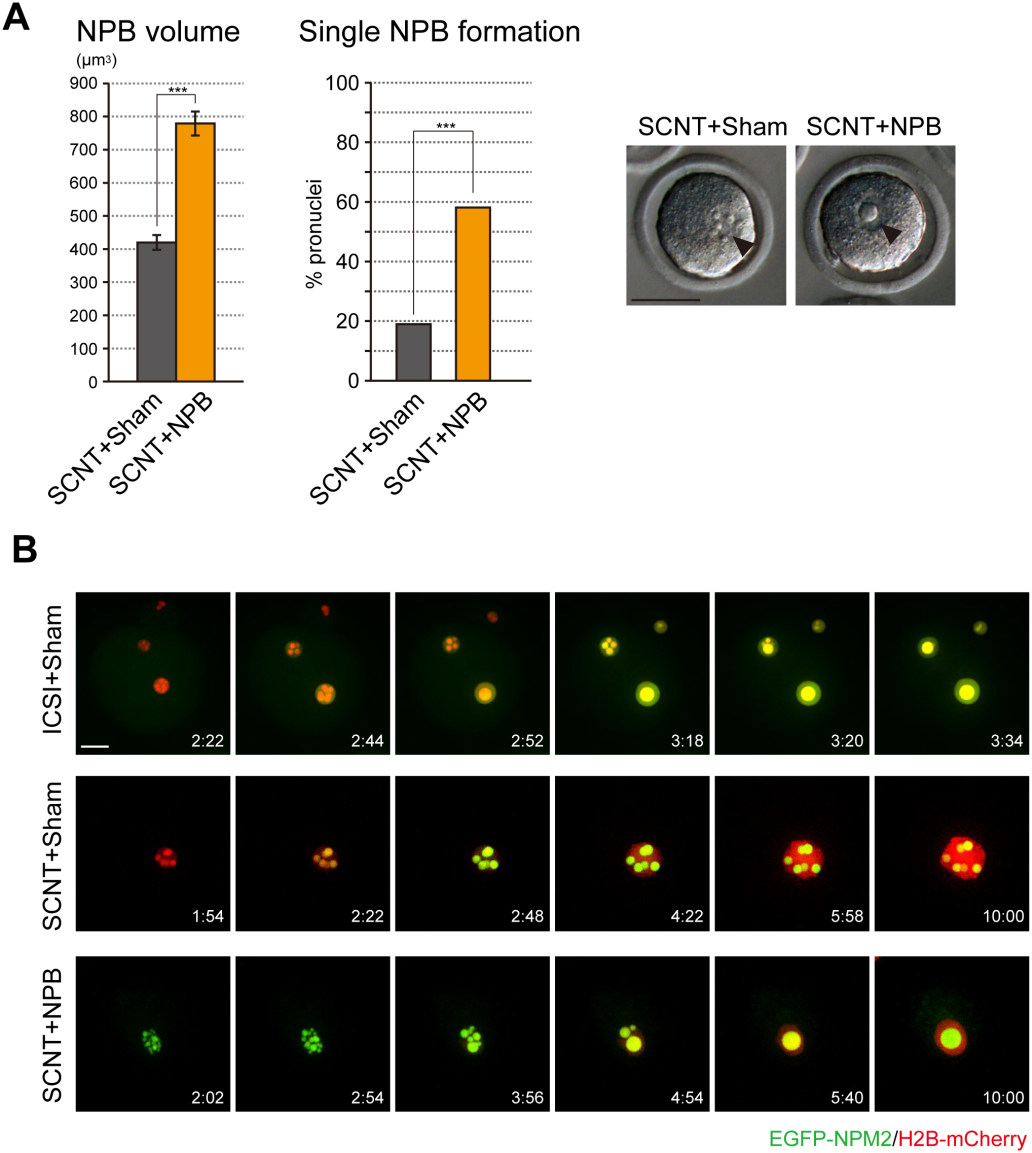
NPB formation in SCNT embryos. (A) NPB volume and single NPB formation in extra-NPB-injected SCNT embryos (SCNT+NPB). The total volumes of NPBs were analyzed in sham-operated SCNT embryos (SCNT+Sham: n = 67) and SCNT+NPB embryos (n = 56). Bars represent the mean ± SEM. Two-tailed, unpaired Student’s *t*-test was performed. The percentages of pseudo-pronuclei with single NPB formation were analyzed in SCNT+Sham embryos (n = 253 pronuclei) and SCNT+NPB embryos (n = 356 pronuclei). Chi-square test was performed. ***P<0.001. Arrowheads indicate NPBs in pseudo-pronuclei. The scale bar is 50 μm. (B) Time-lapse images of NPB formation in the extra-NPB-injected SCNT embryos. Live-cell imaging was performed after injection with *EGFP-NPM2* (green) and *H2B-mCherry* (red) mRNA into oocytes at MII followed by the NPB injection and SCNT. Time after start of imaging (h:mm). The scale bar is 20 μm.

We next examined the NPB formation of somatic cell nuclear transfer (SCNT) embryos that formed multiple NPBs in the pseudo-pronuclei. We considered that the multiple NPB maintenance may be caused by an insufficient volume of NPB, and thus examined whether extra-NPB led to single NPB formation in SCNT embryos. We found that the number of NPBs in the pseudo-pronucleus was decreased by injection of extra-NPBs (SCNT+NPB) compared with sham-operated embryos (SCNT+Sham) (Fig 3A). Moreover, the total volume of NPBs in pseudo-pronuclei of SCNT+NPB embryos became significantly larger than that of control embryos (Fig 3A; SCNT+Sham: 420.0±22.3μm^3^; SCNT+NPB: 778.9±36.1μm^3^). To more thoroughly investigate the formation of NPBs, we conducted live-cell imaging after injection with extra-NPBs and *EGFP-NPM2* mRNA (Fig 3B and S2 Movie). NPM2 is an oocyte-specific nuclear protein and a component of NPBs [24, 25]. During the pronuclear formation in ICSI embryos, a few small NPBs were formed and fused to a single NPB (Fig 3B and S1 Movie). Similar to ICSI embryos, SCNT+Sham embryos formed multiple NPBs at the beginning of pseudo-pronuclear formation (Fig 3B and S2 Movie). However, the multiple NPBs were maintained just before the first cleavage. On the other hand, the SCNT embryos injected with extra-NPBs (SCNT+NPB) formed multiple NPBs, which fused into a single NPB.

### Development of SCNT embryos injected with extra-NPBs

**Fig 4:**
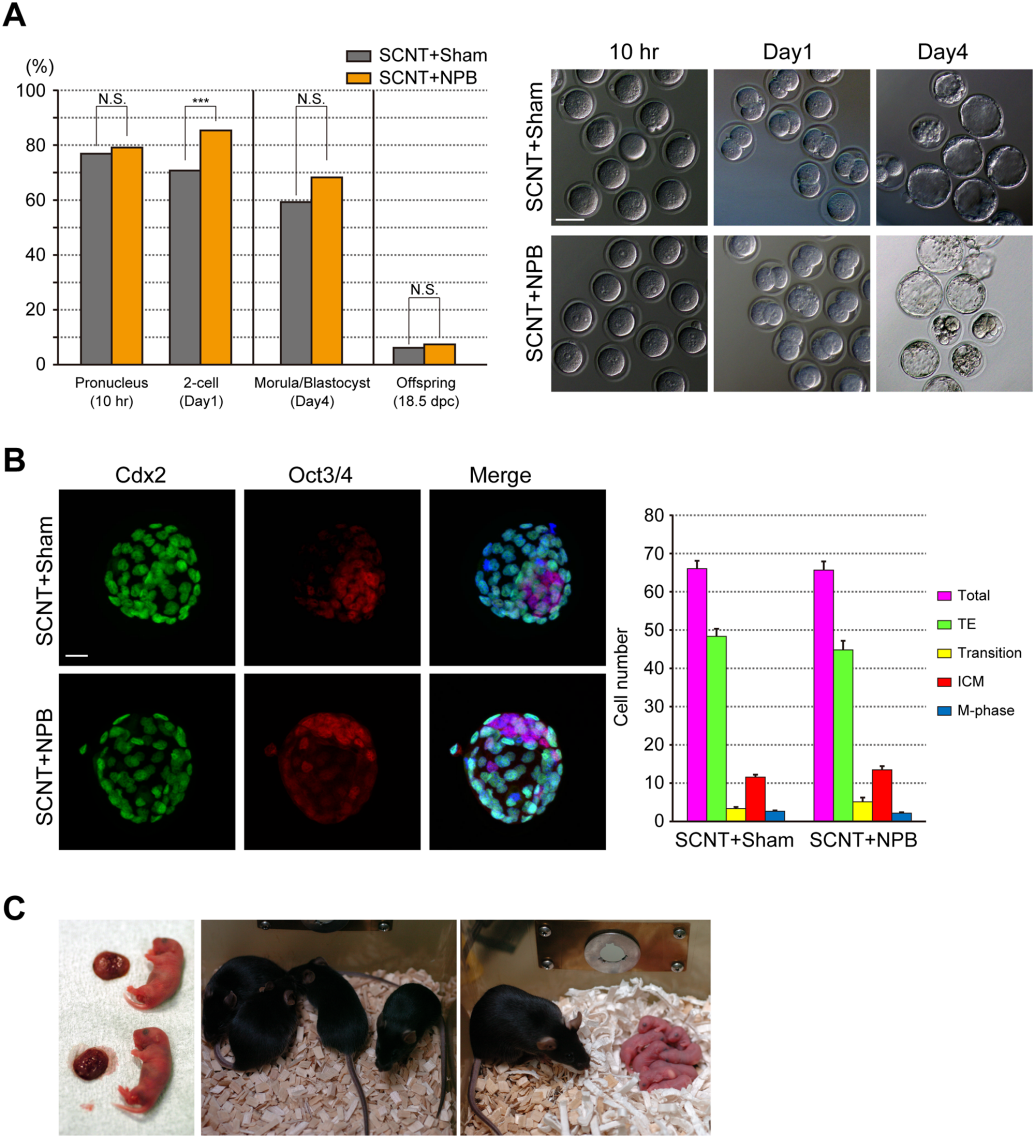
Development of SCNT embryos injected with extra-NPBs. (A) *In vitro* and *in vivo* development of SCNT embryos injected with extra-NPBs (SCNT+NPB). The rates of embryos that formed pseudo-pronuclei, developed to the two-cell stage (SCNT+Sham: n = 432 embryos; SCNT+NPB: n = 467 embryos), and developed to the morula/blastocyst stage (SCNT+Sham: n = 123 embryos; SCNT+NPB: n = 108 embryos) were analyzed. The rate of full-term development was analyzed after the transfer of two-cell-stage embryos into surrogate mothers (SCNT+Sham: n = 189/19 embryos/recipients; SCNT+NPB: n = 178/14 embryos/recipients). Chi-square test was performed. not significant (N.S.). ***P<0.001. The scale bar is 50 μm. (B) Cell numbers and cell types of blastocysts were compared between SCNT+Sham (n = 51) and SCNT+NPB embryos (n = 40). Cell numbers of the trophectoderm (TE) and inner cell mass (ICM) were counted after immunostaining for Cdx2 (green) and Oct3/4 (red), respectively. DAPI staining marks chromatin in blue. Cdx2/Oct3/4 doubly positive cells (Transition) were counted on merged images. Metaphase cells (DAPI staining only) were counted on triple-merged images. The scale bar is 20 μm. Error Bars in the graph indicate the standard error of the mean. Two-tailed, unpaired Student’s *t*-test was performed. (C) Cloned offspring with the placenta (Left) and adult cloned offspring originating (Center) from SCNT+NPB embryos using the same donor cells. An adult offspring from SCNT+NPB embryos with full fertility is also shown (Right).

Because the multiple NPB maintenance in SCNT embryos was reduced by extra-NPB injection, we next examined the development of SCNT embryos that were injected with extra-NPBs (SCNT+NPB). The percentage of SCNT+NPB embryos that formed pronuclei 10 h after strontium activation (79.2%) was similar to that of SCNT+Sham embryos (76.9%, Fig 4A). We selected the embryos forming pseudo-pronuclei to examine their further development. The percentage of SCNT+NPB embryos cleaved 1 day after strontium activation (85.4%) was significantly increased compared with that in SCNT+Sham embryos (70.8%, Fig 4A). The total percentages of SCNT+NPB embryos that developed to morulae and blastocysts were not statistically different but tended to be increased in SCNT+NPB embryos because the cleavage rate was increased (SCNT+Sham: 59.3%; SCNT+NPB: 68.3%; Fig 4A). The morphology of developing embryos was similar between SCNT+Sham and SCNT+NPB embryos (Fig 4A). The numbers of ICM and TE cells in SCNT+NPB blastocysts were similar to those in SCNT+Sham blastocysts (Fig 4B). Moreover, after the transfer of two-cell-stage embryos, we examined the *in vivo* development of SCNT+NPB embryos (Fig 4A). Between SCNT+Sham and SCNT+NPB embryos, there were no significant differences in the final birth rate per transferred embryo (SCNT+Sham: 6.2%; SCNT+NPB: 7.4%). All of offspring grew into adults with full fertility (Fig 4C). These data indicate that extra-NPB injection improves only the cleavage rate of SCNT embryos.

## Discussion

The volume of NPBs in pronuclei was increased approximately twofold by the extra-NPB injection. Previous studies have shown that NPBs injected into previously enucleolated and matured oocyte cytoplasm disassembled and disappear in the oocyte cytoplasm, but after fertilization NPBs are formed in the pronuclei of zygotes [3, 9]. This indicated that NPB materials were scatted and stocked into the cytoplasm until pronuclei formation. Since oocyte NPBs disassembled during oocyte maturation and were reassembled in the pronuclei of zygotes, we considered that the injected extra-NPBs that disassembled in the oocyte cytoplasm at the MII stage were reassembled into NPBs in pronuclei together with the original oocyte NPBs in the present experiments. We also showed that the total volume of NPBs was maintained irrespective of the number of pronuclei. Our previous report showed that the average NPB volume in each blastomere of two-cell embryos was half of the oocyte and zygote NPB volume [8]. This indicates that the total NPB volume per oocyte/embryo was maintained at least until the two-cell stage. Together with the results of the present study, this suggests that the amount of NPB materials plays a major role in the NPB scaling through a limiting component mechanism in zygotes.

The amount of NPB materials also determined whether a single NPB or multiple NPBs formed in the pronucleus. Extra NPB- and extra MII spindle-injection experiments demonstrated that the ratio of pronucleus/NPB is important for single NPB formation. Parthenogenetic embryos formed a single NPB in each pronucleus, but the parthenogenetic embryos that had been injected with an extra-MII spindle formed four pronuclei with multiple NPBs, and the single NPB formation was rescued by extra-NPB injection in these embryos. Some reports suggest that NPBs in pronuclei are a marker of developmental potential in human zygotes [13, 14]. During the pronuclear formation, a few small NPBs are formed and fused to a single NPB along with genome reprograming and chromatin remodeling [26]. In this step, the pronucleus might require a sufficient amount of NPB materials to accomplish the task. Multiple NPB maintenance is sometimes observed in the paternal pronucleus in in vitro-fertilized embryos at the late one-cell stage. Sperm chromatin might require a longer time for remodeling. A previous report showed that NPM2, a component of oocyte NPBs, is one of the factors responsible for sperm chromatin decondensation [26, 27]. However, the mechanism of single NPB formation was still unclear. Some reports showed that the phase separation is a key mechanism underlying the formation of membrane-less organelles such as stress granules in mammalian cells [28], P bodies in C. elegans embryos [29] and nucleoli of Xenopus oocytes [6] and mammalian cells [30]. Phase separation mediates the condensation of nucleic acids and proteins into liquid-like droplets from the surrounding environments, such as through changes in metabolite concentrations or pH changes combined with molecular crowding [31–34]. Phase separation can be achieved through nuclear polymerization and can also be mediated through liquid-liquid phase transition [35, 36], resulting in liquid droplet formation [37]. In liquid-like systems that form droplets, droplet fusion process was occurred through the two mechanisms that diffusion-limited Ostwald ripening and Brownian motion-induced coalescence [29]. Their physical force parameters were also mediated by the surrounding environments. After the pronuclear membrane formation, pronuclei imported nuclear materials such as reprograming factors and NPB materials; result in their environment was changed during pronuclei formation [26]. These reports let us to speculate that the surrounding environment and concentrations of materials were important for single NPB formation.

In cloned embryos produced by SCNT, multiple NPB maintenance in pseudo-pronuclei is one of the apparent abnormalities regardless of mouse strains [15, 38]. The present study has demonstrated that extra-NPB injection reduces the number of NPBs and promotes single NPB formation in pseudo-pronuclei in SCNT embryos. These results are also consistent with our speculation that the amount of NPB materials regulates the single NPB formation. Moreover, the present study has demonstrated that extra-NPB injection improves the cleavage rate in SCNT embryos. However, our results do not clarify the function of NPBs. Previous studies have shown that NPBs are essential for early embryonic development, but they are required only during the early to late pronuclear stage [4, 5]. Several reports have shown that embryos successfully activate rRNA transcription and synthesize proteins without NPBs, suggesting that the NPB has not so much effect on ribosome biogenesis [3, 39]. However, one can speculate as to some important functions of NPBs during the early to late pronuclear stage. In this stage, paternal and maternal chromosomes are dynamically changed and their heterochromatin are associated with NPBs [40]. A recent report showed that a single nucleolar protein, NPM2, could reconstitute NPBs; moreover, mouse NPM2 is required for centromeric/pericentromeric chromatin organization [41]. In the same report, the authors also showed that NPM2-based pericentromeric heterochromatin organization was required for correct segregation of chromosomes [41]. Perhaps for these reasons, the first mitosis was delayed and cleavage rate was decreased in enucleolated or NPM2-knockout embryos [24, 41]. Our present study also showed that the cleavage rate of SCNT embryos was significantly increased by extra-NPB injection. A previous study reported that more than 90% of SCNT embryos exhibited abnormal chromosomal segregation before they developed to the 8-cell stage, as well as severely inhibited post-implantation development [42]. It may be hypothesized that SCNT embryos require a greater volume of NPBs, although the volume of NPBs in fertilized zygotes is sufficient for chromatin remodeling and reprogramming, because the SCNT embryos require full reprogramming the genomes and chromatin of the donor somatic cell. However, the present results showed that the rate of full-term development was still low in the extra-NPB-injected embryos. This might be due to the severe epigenetic abnormalities of SCNT embryos at the pseudo-pronuclear stage [43–47].

## Supporting information

S1 Fig High viscosity medium maintains the structure of NPBs for a long time. (A) Isolated NPBs were disassembled in the culture medium. The scale bar is 50 μm. (B) The NPB structure could be maintained in the high viscosity medium. The scale bar is 50 μm. (C) An NPB was injected into each MII oocyte. Arrows indicate the injected NPB.
(TIF)

S1 Movie Live-cell imaging of NPB fusion during pronuclear formation of an ICSI embryo. Maximum intensity Z-projection images of EGFP-NPM2 (green, NPB) and H2B-mCherry (red, chromosomes) are shown. The time after the start of imaging (h:mm) is shown. The scale bar is 20 μm.
(AVI)

S2 Movie Live-cell imaging of NPB fusion during pseudo-pronuclear formation of extra-NPB-injected SCNT embryos. Maximum intensity Z-projection images of EGFP-NPM2 (green, NPB) and H2B-mCherry (red, chromosomes) are shown. The time after the start of imaging (h:mm) is shown. The scale bar is 20 μm.
(AVI)
