# Supplementary figures and images for: Single nucleolus precursor body formation in the pronucleus of mouse zygotes and SCNT embryos
Source: PLoS One. 2018 Aug 20;13(8):e0202663. doi: 10.1371/journal.pone.0202663 (PMC6101414; doi:10.1371/journal.pone.0202663)

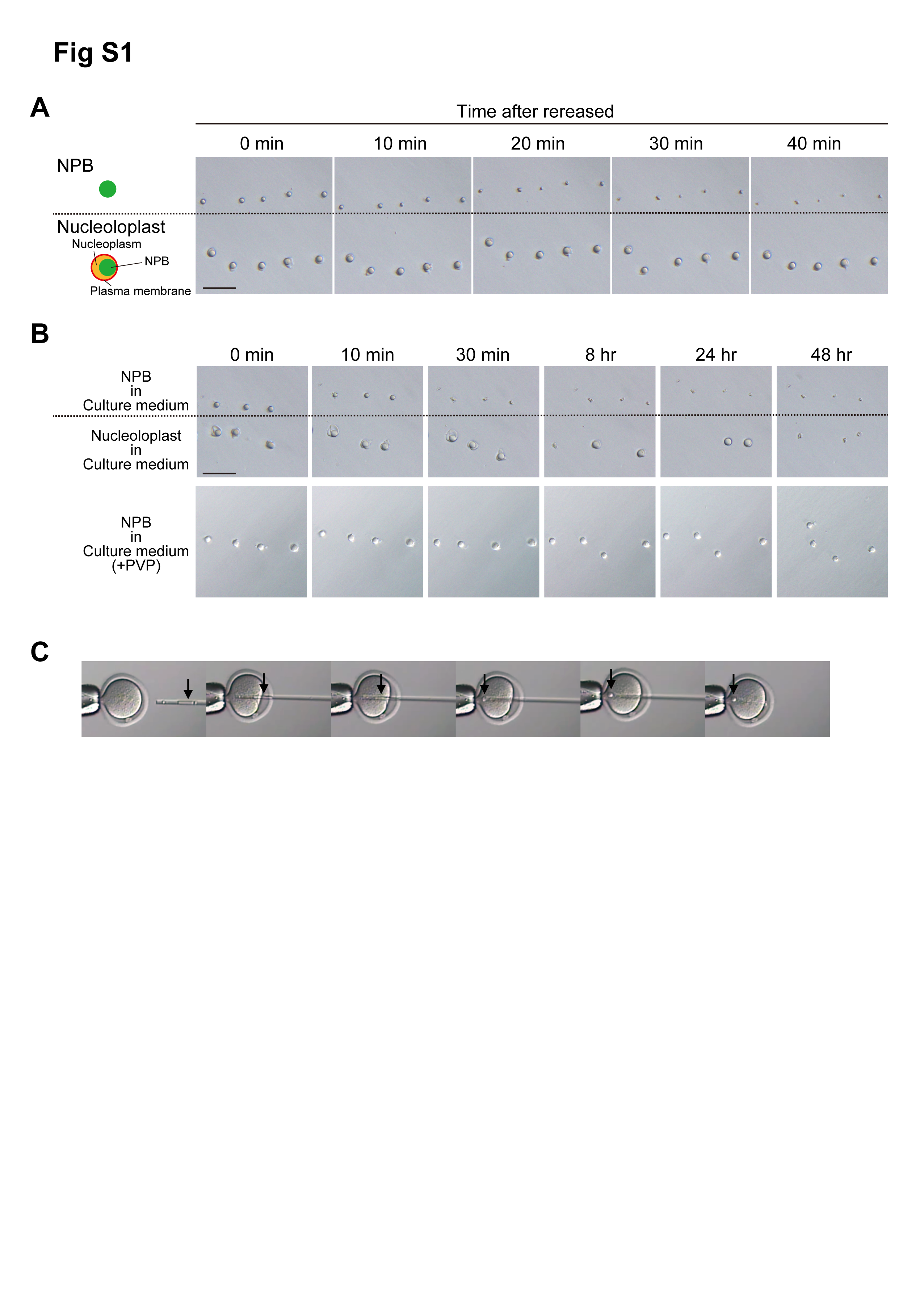

Supplement: S1 Fig — (A) Isolated NPBs were disassembled in the culture medium. The scale bar is 50 μm. (B) The NPB structure could be maintained in the high viscosity medium. The scale bar is 50 μm. (C) An NPB was injected into each MII oocyte. Arrows indicate the injected NPB. (TIF) [file pone.0202663.s001.tif]
